# Supplementary material for: Technological Characterization of Lactic Acid Bacteria Strains for Potential Use in Cheese Manufacture
Source: Foods. 2023 Mar 9;12(6):1154. doi: 10.3390/foods12061154 (PMC10048630; doi:10.3390/foods12061154)
Supplement: Supplementary file 1 [file foods-12-01154-s001.zip › foods-2201510-supplementary.pdf]

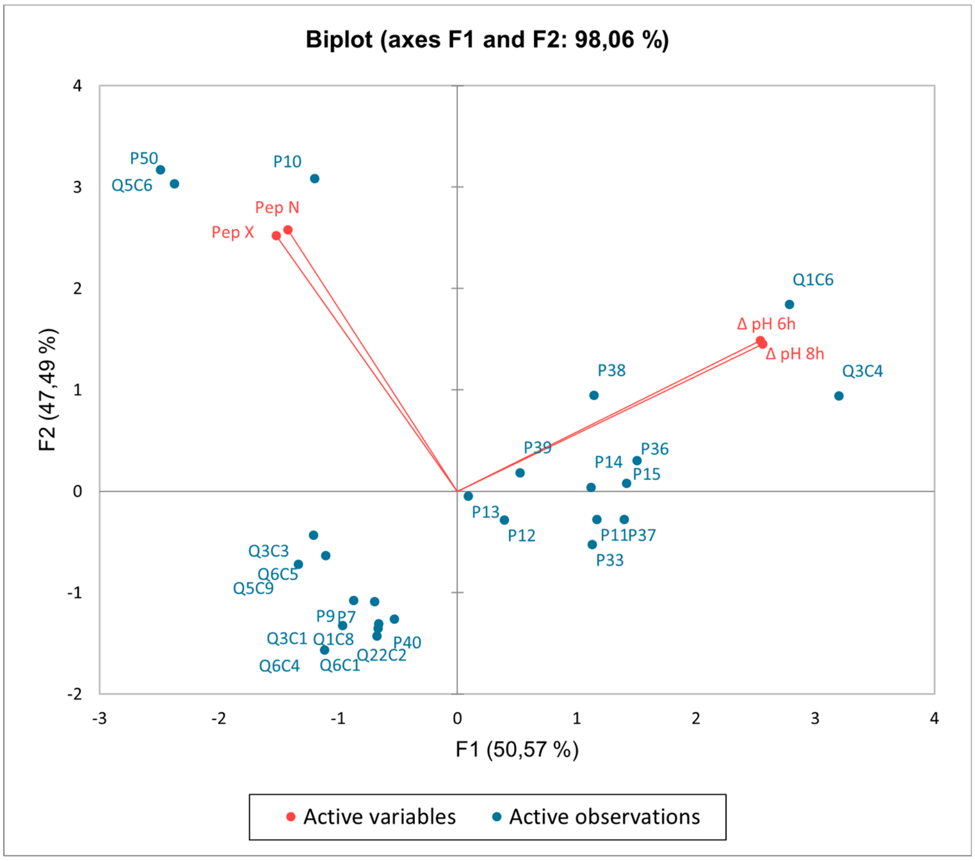


**Figure S1:** Biplot of the principal components analysis (PCA) of LAB strains isolated from Brazilian and Italian cheeses in relation to aminopeptidase activities (Pep N and Pep X) and acidifying activity (ΔpH 6h and ΔpH 8h).
